# A new ultradian rhythm in mammalian cell dry mass observed by holography

**DOI:** 10.1038/s41598-020-79661-9

**Published:** 2021-01-14

**Authors:** Lamya Ghenim, Cédric Allier, Patricia Obeid, Lionel Hervé, Jean-Yves Fortin, Maxim Balakirev, Xavier Gidrol

**Affiliations:** 1 Univ. Grenoble Alpes, CNRS, CEA, INSERM, IRIG, Biomics, 38000 Grenoble, France; 2 Univ. Grenoble Alpes, CEA, INSERM, IRIG, Biomics, 38000 Grenoble, France; 3 Univ. Grenoble Alpes, CEA, LETI, 38054 Grenoble, France; 4 Laboratoire de Physique et Chimie Théoriques CNRS UMR7019, Université de Lorraine, 1 Boulevard des Aiguillettes, BP 70239, 54506 Vandoeuvre-lès-Nancy Cedex, France

**Keywords:** Oscillators, Proteasome

## Abstract

We have discovered a new 4 h ultradian rhythm that occurs during the interphase of the cell cycle in a wide range of individual mammalian cells, including both primary and transformed cells. The rhythm was detected by holographic lens-free microscopy that follows the histories of the dry mass of thousands of single live cells simultaneously, each at a resolution of five minutes. It was vital that the rhythm was observed in inherently heterogeneous cell populations, thus eliminating synchronization and labeling bias. The rhythm is independent of circadian rhythm, and is temperature-compensated. We show that the amplitude of the fundamental frequency provides a way to quantify the effects of, chemical reagents on cells, thus shedding light on its mechanism. The rhythm is suppressed by proteostasis disruptors and is detected only in proliferating cells, suggesting that it represents a massive degradation and re-synthesis of protein every 4 h in growing cells.

## Introduction

Spatiotemporal order in a living system is believed to be a result of nonlinear chemical reactions and high variability. This leads to the generation of periodic oscillations that are characterized by a central timekeeping mechanism, which is defined as a clock for biological processes^1^. These rhythms allow organisms to adaptively cope with changing environments, such as the circadian clock imposed by Earth, thus conferring robustness and a competitive advantage. Many periodic biological processes have been described such as circadian rhythms, ultradian rhythms (periods shorter than a day) and the cell cycle. Some of the processes are endogenous, whereas others may be driven by external cues at the level of the organism. Nevertheless, the basic pacemaker of biological clocks appears to reside on a cellular level and is integrated into molecular networks of the cell. Studies in cultured cells have significantly advanced our understanding of the molecular mechanisms of the cell cycle and, more recently, of other cell-autonomous biological clocks. To detect periodicity in cell behavior, the expression of specific genes is usually measured by biochemical sampling or by using specific luminescent markers. Because the individual rhythms in a cell population are out of phase, they need to be synchronized by using an external stimulus. As a result, the most common approaches to studying biological clocks have a poor time resolution and may suffer from artifacts introduced by labeling or synchronization. On the other hand, our ability to study single-cell dynamics in asynchronous culture has been limited by the availability of quantitative phase imaging techniques for simultaneous, time-resolved, single-cell data acquisition from thousands of cells in parallel. Large population sampling is required to overcome the complex "noisy" behavior of a single cell and determine the standard characteristics of cell dynamics.

**Figure 1:**
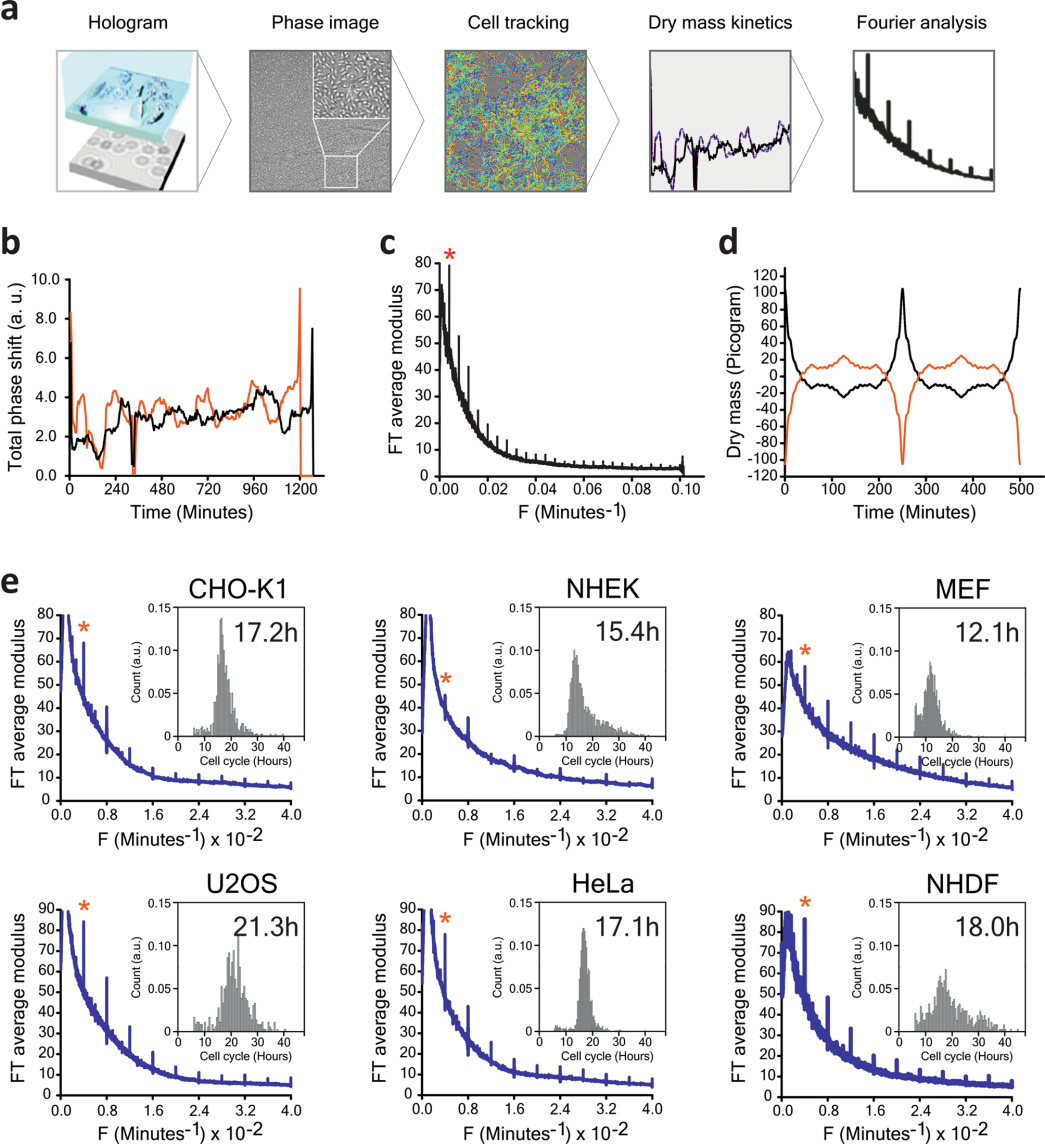
A new ultradian rhythm in mammalian cells. (**a**) Schematic view of the experimental approach (see “Materials and methods” for details). (**b**) Individual histories of the dry mass of two individual mouse embryonic fibroblast (MEF) cells are shown during the cell cycle interphase. (**c**) Fourier amplitudes of the dry mass traces as a function of frequency averaged over 702 MEF cells. First sharp peak (asterisk) of the dominant frequency of 0.004 min^−1^ along with its higher harmonics is clearly distinguished from the background 1/f noise. (**d**) Reconstruction of the signal of dry mass (pg) by means of an inverse Fourier transform (two possible solutions). (**e**) The 4 h rhythm observed in various mammalian cell lines. The inserts show the distributions of the cell cycle lengths, the median cell cycle length is indicated in the insert.

Recently, we have described a lens-free microscopy technique that allows real-time measurements of dry mass with a precision of approximately 35 pg. It is based on holographic interferences between the optical path that are propagated through a pinhole and waves scattered by the cell. The dry mass of the cell is calculated by integrating the phase of the diffracted light over the whole area of the cell^2^. Compared to conventional optical methods, lens-free microscopy provides a unique way to track thousands of live cells in real time and with a large field of view (30 mm^2^) without any labeling or synchronization. In the present work, we use this technique to demonstrate periodic oscillations in the cell dry mass (Fig. 1a). In the raw signal these are superimposed on a linear background that reflects cell growth.

## Results

We tracked thousands of live cells, taking images every 5 min over several days without labeling or synchronization. The reconstructed phase signals for individual cells were used for cell segmentation and calculation of the cell dry mass (Supplementary Figs. S1–S4)^2^. Mitotic events and the cell cycle duration were determined from abrupt changes in the cell dry mass concomitant with the appearance of a new cell trajectory (Supplementary Fig. S3). Cell dry mass dynamics were analyzed during the interphase, from the first detected mitotic event until the following, or until the track was lost. For each cell, we computed the Fourier transform (FT) of the dry mass change during the interphase trajectory. The averaged absolute amplitude was normalized by trace length.

The dry mass history in mouse embryonic fibroblasts (MEFs) revealed a dominant frequency of 0.004 min^−1^, and higher harmonics, superimposed on 1/f noise (Figs. 1b,c). Reconstruction of the signal by applying an inverse Fourier transform to exclude the noise, reveals periodic symmetric spikes, 30 min wide, every 4.17 h (SD = 0.0039 h) (Fig. 1d and Supplementary Fig. S5). In Hela, U2OS, MEF, CHO-K1, NHEK and NHDF cells we also observed the same principal frequency of 0.004 min^−1^ (Fig. 1e). The amplitude of the dry mass change was approximately 100 pg in MEF cells; three times the method precision (Fig. 1d). Examination of experimental conditions and analysis of a random dataset ruled out artifacts (Supplementary Figs. S6a,c). Similarly, no oscillations were found when the cells were fixed (Supplementary Fig. S6b). The frequency emerged from 1/f noise only when more than 100 living cells were analyzed (Supplementary Fig. S6b).

### Role of cell cycle and circadian oscillators

**Figure 2:**
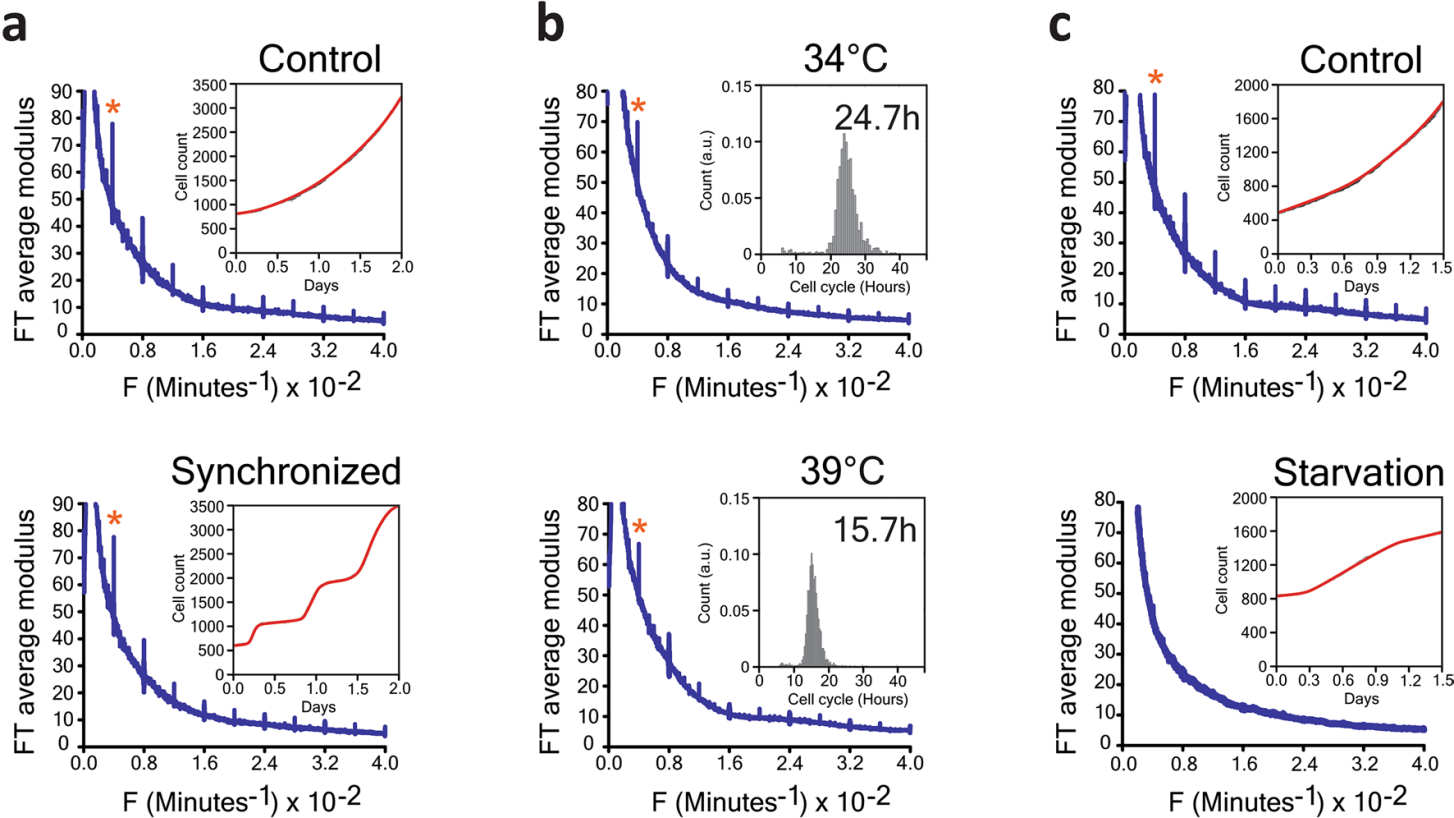
Effect of the cell cycle manipulation on the rhythm. (**a**) Synchronization of HeLa cells using a double thymidine block does not affect the rhythm. The inserts show cell growth curves for the control and synchronized cells plotted using the data from direct cell counts by lens-free microscopy. (**b**) Comparison of HeLa cells at 34 °C (top) and 39 °C (bottom). The characteristic frequency of the rhythm does not change despite the significant change in the cell cycle length (inserts). (**c**) Serum starvation eliminates the 4 h rhythm. The inserts show cell counts in control (top) and serum starvation (bottom) conditions.

**Figure 3:**
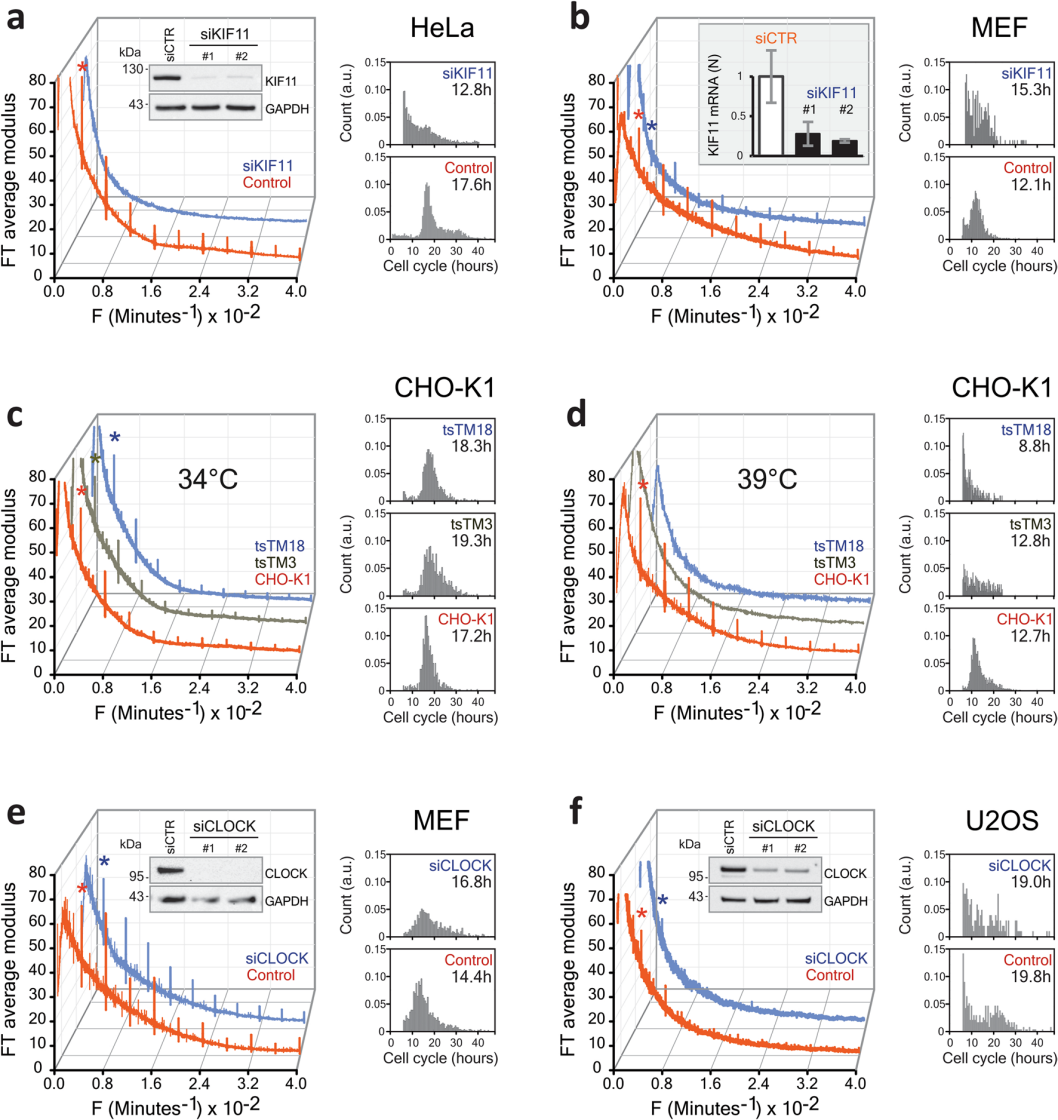
Relationship between the rhythm, cell cycle and circadian clock. (**a**) Cell cycle arrest by siKIF11 treatment in HeLa cells suppresses the 4 h rhythm. Western blots (in insert) confirm the inhibition of KIF11 expression (the effect of two specific siRNAs is compared to non-specific siCTR). (**b**) Incomplete cell cycle arrest by siKIF11 in MEF cells does not suppress the 4 h rhythm. Quantitative PCR analysis (in insert) shows the inhibition of KIF11 expression by two specific siRNAs in MEF. To the right of (**a**) and (**b**) we show the distributions of the cell cycle lengths corresponding to the two data sets. (**d**) Effects of cell cycle inhibition in temperature-sensitive CHO-K1 mutants at 39 °C, compared to the permissive 34 °C temperature (**c**). Cell cycle arrest in temperature-sensitive CHO-K1 mutants at 39 °C suppresses the 4 h rhythm. To the right of (**c**) and (**d**) we show the distributions of the cell cycle lengths for the three cell types. (**c**) Effect of circadian rhythm inhibition by siCLOCK in (**e**) MEF and (**f**) U2OS. Knocking down CLOCK does not eliminate the rhythm in MEF and U2OS. The Western blots (see inserts) show the inhibition of CLOCK expression by siCLOCK 1 and 2 in MEF and U2OS cells. To the right of (**e**) and (**f**) we show the corresponding distributions of the cell cycle lengths.

So far, we have seen that the 4 h rhythm in cell dry mass seems to be general in mammalian cells. A priori, this could be a distinct ultradian rhythm or simply a harmonic of a longer-period biological clock such as the circadian rhythm or the cell cycle. We therefore investigated potential links between the 4 h rhythm and these oscillators. Synchronization did not significantly change the period or the amplitude of the rhythm (Fig. 2a). Despite the variability in cell cycle duration, each cell line showed the same oscillation period suggesting the rhythm was not a cell-cycle harmonic (Fig. 1e). Furthermore, varying the temperature from 34 to 39 °C significantly influenced the cell cycle length in HeLa and CHO-K1 cells, while leaving the period of dry mass oscillation unchanged (Figs. 2b, 3c,d). Thus, unlike the cell cycle, the rhythm showed perfect temperature compensation, a hallmark of a biological clock^3–6^. We used serum starvation to induce G0/G1 cell cycle arrest in HeLa cells. Over 48 h the cells, while still alive, gradually ceased proliferating and the rhythm disappeared (Fig. 2c). For HeLa cells arrested in the G2/M phase by knocking down the KIF11/EG5 gene, the rhythm was also suppressed (Fig. 3a, Supplementary Fig. S7 and Supplementary Movie S1). By contrast, in MEF cells, the mitotic arrest induced by KIF11 knockdown was not as efficient and the rhythm remained (Fig. 3b and Supplementary Movie S2). Next, we examined the temperature-sensitive CHO-K1 cell lines tsTM3 and tsTM18, which carry mutations in ubiquitin-activating enzyme UBA1 and replication regulator SMU1, respectively, and are known to undergo S-G2 or S and G2 cell cycle arrests at the non-permissive temperature of 39 °C^7,8^. We found that, contrary to wild-type CHO-K1 cells, both mutants displayed the 4 h rhythm only at the permissive temperature of 34 °C (Fig. 3c,d). Therefore, while not a direct consequence of the cell cycle, the oscillations are a feature of proliferating cells. Finally, to address the role of circadian rhythms we knocked down CLOCK, a key circadian regulator, in MEF and U2OS cells, and found no effect on the oscillations (Fig. 3e,f and Supplementary Fig. S7). Thus, despite being temperature-compensated and an integer submultiple of 24 h, the 4 h rhythm does not seem to be a circadian harmonic. Taken together, these results strongly suggest that the periodic changes in cell dry mass represent a new ultradian rhythm in growing cells.

### Role of cell machineries

**Figure 4:**
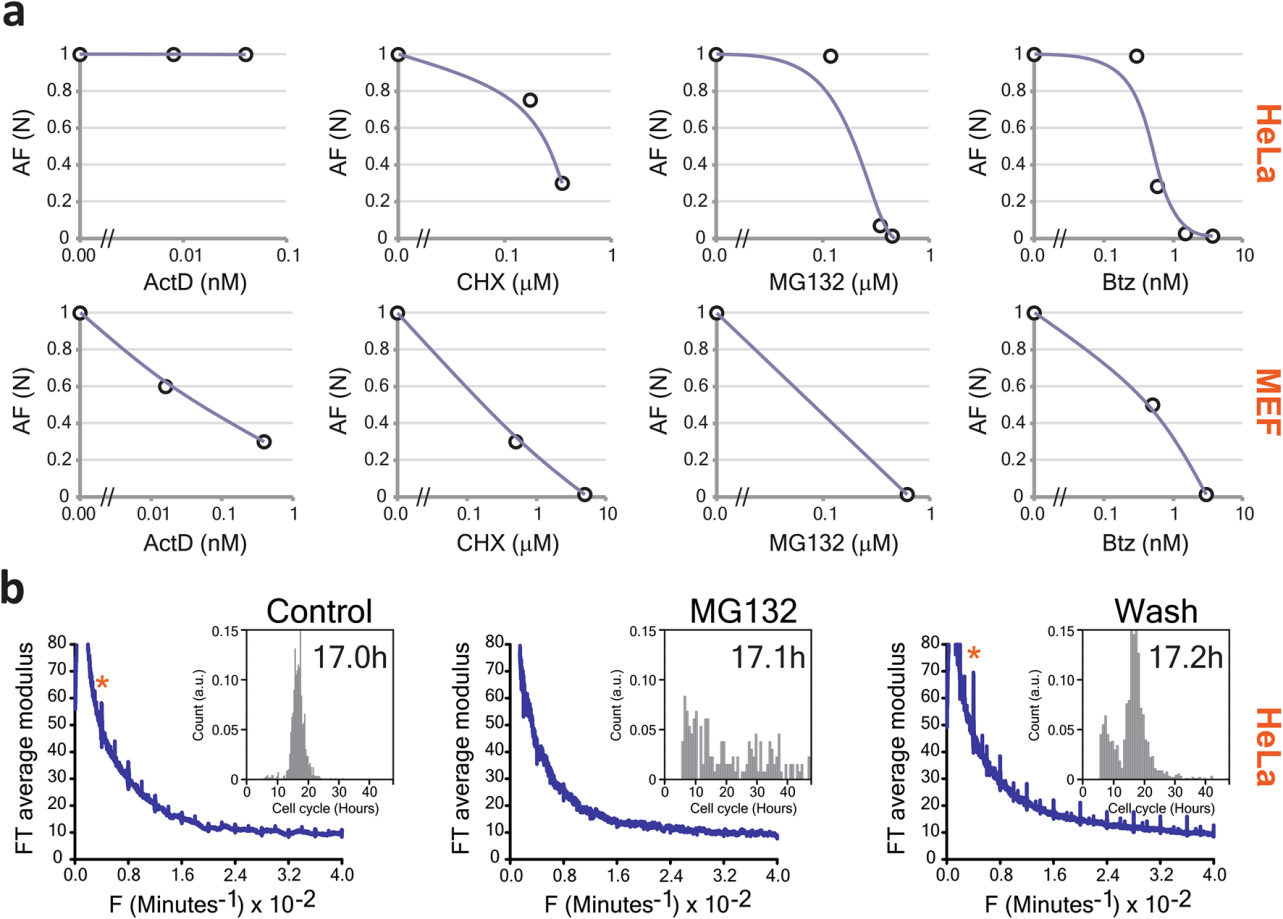
Effect of different inhibitors on the rhythm. (**a**) Effect of different inhibitors on the rhythm measured by the amplitude of the fundamental peak at 0.004 min^−1^ in Hela (first row) and MEF (second row). (**b**) Inhibition of the 4 h rhythm by MG132 is reversible. Compared to control conditions (left panel), treatment of HeLa cells with 0.45 μM of MG132 for 24 h completely suppressed the rhythm (middle panel). Subsequent removal of the MG132 by rinsing restored the oscillations with increased amplitude (right panel). The inserts show the distributions of the cell cycle lengths.

Proteins represent more than half of the cell dry mass, followed by nucleic acids (~ 15%), lipids (~ 10%) and sugars^9,10^. The periodic change in the dry mass might result from the synthesis/degradation of these molecules or from their transport in or out of the cell. To obtain more information on molecular mechanisms, we used: (i) actinomycin D (ActD) to inhibit transcription, (ii) cycloheximide (CHX) to inhibit protein synthesis, and (iii) proteasome inhibitors MG132 and bortezomib (Btz) to block ubiquitin-dependent protein degradation. To analyze the drug response, we used the average Fourier amplitude of the fundamental peak (AF) to define the oscillator strength. This parameter showed very little variability within a cell batch, providing a reliable and quantitative measure of the inhibition effect (Supplementary Fig. S8). Since the rhythm appears in proliferating cells (Figs. 2c, 3a,c,d), its inhibition by drugs could be either direct, or through the cell cycle arrest. To exclude the latter, we analyzed only cell trajectories between two mitotic events. The drugs decreased the oscillator strength in MEF and HeLa cells, while the periodicity was unchanged (Fig. 4a). Transcription inhibition with ActD failed to arrest the 4 h rhythm in MEF cells, even at the highest dose that induced ~ 50% cell mortality (Fig. 4a and Supplementary Fig. S9). In contrast, inhibition of either protein synthesis (CHX) or degradation (MG132 and Btz) completely suppressed the rhythm with relatively low cytotoxicity (Fig. 4a and Supplementary Fig. S9). Similarly, in HeLa cells, virtually nontoxic doses of CHX or Btz eliminated the 4 h rhythm, while ActD showed no significant effect (Fig. 4a). MG132 also arrested the rhythm in HeLa cells albeit with significant cytotoxicity (~ 50% cell death, Supplementary Fig. S9). Therefore, the oscillations of the cell dry mass are particularly sensitive to proteostasis inhibition and may represent changes in protein level during cell growth. The effect of Btz was the most remarkable; the rhythm is already suppressed at 0.6 nM, the Ki value for the 20S proteasome (Fig. 4a)^11^. The removal of proteasome inhibitor restored the rhythm with, strikingly, an increased amplitude: the transient proteasome inhibition must either increase the amplitude of protein pulses or induce more cells to oscillate (Fig. 4b). We argue therefore that the proteasome is one of the main molecular machineries regulating the 4 h ultradian rhythm.

## Discussion

Periodic changes in protein levels have been shown previously; the best known example is the oscillations of the key regulators of the cell cycle^9,12,13^. Recent proteomics studies suggested that the abundance of more than 10% of cellular proteins are subject to the 24 h circadian rhythm^14^. Higher frequency ultradian oscillations have also been reported. In the early 1960s, Brodsky and collaborators, by analyzing the incorporation of radioactively labeled amino acids into cellular proteins, suggested a periodicity of one to two hours in the protein synthesis rate^15^. The same authors have reported the observation of ultradian dry mass oscillation in retinal ganglion cells, as observed by interferometry^15^. In the last decade, ultradian oscillators originating in transcriptional-translational-posttranslational feedback loops have been discovered for transcription factors p53, Hes1, and NFkB^16–18^*.* Notably, there was a change in the level of the proteins implicated in these signaling circuits, which have periods between 2 and 6 h. More recently, the oscillations of another transcriptional regulator, XBP1, has been shown to coordinate a new 12 h ultradian rhythm^19^. Very recently, Liu et al.^20^ reported repetitive dips in the coefficient of variation (CV) of the cell growth rate in HeLa cells. The authors used quantitative phase microscopy interferometry to measure the dry mass of the cells during the cell cycle at a 30 min time resolution. They tentatively suggested that the dips in the cell growth rate CV might reflect a novel oscillatory circuit in protein synthesis/degradation that is intrinsic to cell growth rate regulation. Although the reported periodicity was close to 4 h, it was significantly temperature dependent: 4.7 h at 33 °C and 5.8 h at 36 °C.

The 4 h rhythm^21^ we describe here differs, however, in several important ways from the previous observations. First, it appears to be cell-autonomous, robust and universal, as it was found in all cultured mammalian cells we examined. The rhythm is present in asynchronous cell cultures growing in standard conditions, and no additional stimuli are required to trigger it. Second, the 4 h rhythm is not limited to a few specific proteins; rather, it involves global changes in the total mass of cell constituents. Third, the 4 h rhythm is temperature-compensated; this was not the case for the ultradian rhythms mentioned above. Finally, our analysis with the inverse Fourier transform indicates that the 4 h rhythm has a particular nonsinusoidal waveform, where the long delay periods are followed by rapid (~ 30 min) symmetric changes in the cell dry mass (Fig. 1d). This pulsatile dynamics may explain why the rhythm was not observed in previous works that used lower time resolution (> 30 min) in sampling. We also needed to follow hundreds of cells in parallel to begin to see this periodic signal, which required quantitative phase imaging techniques with a large field of vision.

Our results give a first glimpse into the underlying mechanism of the 4 h oscillator. The rhythm disruption by proteasome inhibition and its stimulation upon inhibitor removal suggests that the proteasome is implicated in oscillator regulation. This is not surprising, as the proteasome degrades key pacemaker proteins, meaning that it has an essential role in almost all reported biological rhythms. The universality and the amplitude of the mass oscillations we see (Fig. 1) suggest that the proteasome, by itself, is a 4 h rhythm pacemaker, and its activity is responsible for pulsatile dynamics of the total mass of proteins. The existence of posttranslational proteasome-based oscillators has been predicted previously by mathematical models that comprise both protein synthesis and degradation^22–25^. It should be noted that our analysis cannot determine whether the dry mass is rising during the pulses as a result of increased synthesis or is dropping because of accelerated degradation (Fig. 1d). Even though both possibilities remain, the second hypothesis seems more thermodynamically likely.

Another aspect of the 4 h rhythm is that it is linked to the cell cycle. Curiously, pioneering work by Klevecz suggested that endogenous oscillations in protein synthesis set the generation time of the cell cycle as a multiple of a fundamental 4 h period^26,27^. This ultradian oscillator was found to be temperature compensated^26^. Lloyd and Volkov later proposed a mathematical model for the cell cycle to explain these results. This model included a fast ultradian component with pulsatile dynamics that were similar to what we see in our reconstructed signal^28^. Therefore, the 4 h rhythm we describe here may reflect the same timekeeping mechanism that regulates cell growth during interphase and dictates cell cycle duration. Although we found that the inhibition of the 4 h rhythm with low doses of proteasome inhibitors did not block ongoing cell division, it certainly prevented subsequent mitosis because the cultures finally stopped growing (Supplementary Fig. S10). Furthermore, the suppression of the 4 h rhythm following cell cycle arrest suggests a coupling of the two oscillators. Finally, as the proteasome is the ultimate regulator of cyclin/CDK machinery, our conclusions should be compatible with the current models of cell cycle regulation.

## Materials and methods

### Cells

Neither animal subjects nor human research participants were included or used in this study.

### Cell culture

*HeLa cells* were cultured in high glucose DMEM supplemented with GlutaMAX, pyruvate, and 10% calf serum (Gibco). *NHEK cells* (Lonza) were cultured in KBM-gold Bulletkit (Lonza) and were passaged with the ReagentPackSubculture reagents (Lonza). NHDF-Ad-Der Fibroblasts FGM-2 cells (Lonza) were cultured in FGM-2 BulletKit (Lonza) and were passaged with the ReagentPack Subculture Reagents (Lonza). *U2OS cells* (ATCC) were cultured in McCoy’s 5A medium (ATCC) and 10% calf serum. Mouse Embryonic Fibroblasts (C3H/10T1/2 clone 8, ATCC) were cultured in Eagle’s Minimum Essential Medium (EMEM; ATCC) and supplemented with 10% calf serum. *CHOK1-WT, TSTM3* and TSTM18 (Research Center for Radiation Protection, Chiba, Japan) were cultured in Nutrient Mixture F-12 Ham (SIGMA) supplemented with 1 mM l-glutamine (SIGMA) and 10% calf serum. All media was supplemented with 1% Penicillin/Streptomycin (Invitrogen).

### Cytotoxicity assays: determination of LD50 by dose response

LD50 was determined by testing in duplicate the drugs at different concentrations on cells seeded 24 h, in 96-well plates, prior to drug treatment. CellEvent Caspase-3/7 Green Detection Reagent (Invitrogen, Ref. C10423, C10723) was added, to each well, directly with the drug mixture in a final concentration of 2 µM per well. 24 and 48 h after drug and treatment with the CellEvent Caspase-3/7 reagent, cells were counterstained with Hoechst. Images were acquired using a High Content CellInsight NXT HCA automated microscope (Thermo Scientific). Analysis was performed using R software^29^ and the percentage of dead cell was evaluated based on negative control levels. The real time imaging experiments with cells treated with different drugs were then performed during 48 h at concentrations much below the concentration that induces 50% mortality.

### Drug washout experiments

All drugs were prepared first in DMSO (Sigma-Aldrich). The cells were treated with drugs 24 h after being seeded and grown at 37 °C. To inhibit transcription, Mouse Embryonic Fibroblasts and HeLa cells were treated respectively with 0.016 nM, 0.4 nM and 0.008 nM, 0.04 nM of Actinomycin respectively. To inhibit translation HeLa cells and Mouse Embryonic Fibroblasts were treated respectively with 0.175 µM, 0.35 µM and 0.5 µM, 5 µM of Cycloheximide respectively. To inhibit protein degradation HeLa and MEF were treated respectively with 0.3 nM, 0.6 nM, 3.75 nM, 5 nM and 0.5 nM, 3 nM of Bortezomib. Otherwise, to inhibit protein degradation HeLa and MEF cells were treated respectively with 0.12 µM, 0.35 µM 0.45 µM, and 0.62 µM of MG132. To reverse the inhibition of protein degradation, HeLa cells were washed out three times with a medium without drugs.

### Small interfering RNA transfection

RNAi screens were performed in 6-well plates and three Fibronectin (F1141-5MG from Sigma Aldrich) coated 35 mm glass bottom dishes (ref 150682 Thermo Scientific). RNA interference against EG5 (ref Qiagen Hs_KIF11_6 Cat N°./ID: SI02653693, Mm_Kif11_3 FlexiTube siRNA (5 nmol) Cat N°./ID: SI01082389, Mm_Kif11_2 FlexiTube siRNA Cat No./ID: SI01082382), and against Clock (Mm_Clock_6 FlexiTube siRNA Cat N°./ID: SI02707908; Mm_Clock_2 FlexiTube siRNA Cat N°./ID: SI00168595) were performed with two independent siRNAs from Qiagen each, and each siRNA transfection was repeated two times. As control we used siRNA AllStar negative control (AS) (Qiagen, Ref. 1027281). HeLa and C3H10T1/2 cells (20,000 cells per petri dish and 80,000 cells per well) were seeded or plated in complete medium 24 h before transfection. Transfection of these cells was performed using Lipofectamine RNAiMAX reagent. The RNA-lipid complexes were prepared and then added to the cells. First, Lipofectamine RNAiMAX Reagent was diluted in Opti-MEM Medium, second siRNA was diluted in the same solution. The two mixtures were incubated at room temperature for 10 min. Diluted siRNA was added to the diluted Lipofectamine RNAiMAX Reagent and this mixture was then well homogenized and incubated for 15 min at room temperature. Finally, the siRNA-lipid complex was distributed on the cells. The final concentration of siRNA per well was 1–10 nM. The plates as well as the glass bottom dishes were placed in the incubator at 37 °C for 48 h. The glass bottom dishes were placed on the lens-less microscopes for data acquisition within the incubator. The transfection was stopped after 48 h. The 6-well plates were used for the PCR and western blot experiments as controls of the optical experiments.

### Western Blot analysis

After 48 h in the incubator, cells in the 6-well plates were lysed using RIPA buffer (Sigma, Ref. R0278) supplemented with Complete Mini Protease Inhibitor Cocktail (Sigma, Ref.P8340). The buffer (50 µL) was added to each well and the plate was incubated at 4 °C for 30 min. The lysates are homogenized, collected and put in a 1.5 mL Eppendorf tube at 4 °C for another 15 min. Centrifugation was then done at 13,000 rpm for 15 min at 4 °C and the supernatant was taken and stored at − 20 °C while waiting for the protein dosage. The protein dosage was done according to Pierce BCA Protein Assay Kit protocol (Thermo Scientific, Ref.23227). The range of BCA was made from a commercial solution at 2 mg/mL. Dilutions are made in RIPA. Quantification was done in duplicates with 5 µL samples and 100 µL BCA mix prepared according to the manufacturer’s protocol. The plate was then incubated for 45 min at 37 °C and the reading of the plate was performed using the TECAN Infinite M1000 reader at 562 nm wavelengths. To examine the expression of the target genes, the same amount of total protein was analyzed by western blot using target-specific antibodies. For siEG5 and siClOCK experiments, 7 µg (protein samples) were subjected to polyacrylamide gel electrophoresis on NuPAGE Novex 12% Bis–Tris Protein Gel (Life Technologies, Ref.NP0342BOX) in MES buffer. The separated proteins were transferred onto a nitrocellulose membrane (D. Dutscher, Brumath, France) using iBlot 2 Transfer Stack according to the manufacturer’s protocol. The membranes were blocked in 5% non-fat milk in Tris-buffered saline, 0.1% Tween 20 (TBST, Sigma) for 1 h at room temperature (RT) and incubated with primary antibodies diluted at 1:500 in 5% non-fat milk in TBS-T overnight at 4 °C. We used rabbit polyclonal antibodies directed against *human EG5 (1/1000,* ab171963), rabbit polyclonal antibodies directed against *human and mouse CLOCK *(1/2000, ab93804), mouse polyclonal antibodies against tubuline (1/2500, SIGMA T-6074) and actin (1/5000, (H-196)) Santa Cruz Biotechnology Sc-7210 and rabbit polyclonal Anti-GAPDH [(FL-335) Santa Cruz Biotechnology Sc-25778]. Membranes were washed three times for 10 min in TBS-T and incubated with secondary horseradish peroxidase-conjugated antibodies (anti-mouse or anti-rabbit, eBiosciences, Paris, France) at a dilution of 1:10,000 in TBST for 1 h at room temperature. Membranes were washed three times for 10 min in TBST. Detection was performed with an ECL Prime kit [SuperSignal West Pico PLUS Chemiluminescent Substrate (Thermo Scientific, Ref.34577) and the SuperSignal West Femto Maximum Sensitivity Substrate (Thermo Scientific, Ref.34095) according to the manufacturer’s guidelines]. Chemiluminescence was analyzed using a ChemiDoc Touch Imaging System (BioRad, Marne-la-Coquette, France), and quantification of the intensity of the bands was performed using the ImageLab Touch Software (BioRad).

### Gene expression analysis by RT-qPCR

The commercial RNeasy Plus Mini Kit (Qiagen, Ref.74136) was used for total RNA extraction according to the manufacturer’s instructions. 48 h after the Embryonic Mouse Fibroblasts (C3H/10T1/2 clone 8) (as above for invasion experiment) transfection with the siRNA EG5 (KIF11-2 and KIF11-3), the media was removed and the cells were lysed directly in the wells with 350 µL Buffer RLT supplied by the kit. The total RNA was eluted in 40 µL of RNase-free water and the sample were stored at − 80 °C.

RNA concentration was measured using a Thermo Scientific NanoDrop 1000 Spectrophotometer (Thermo Scientific). Then, 2 µg RNA were reverse-transcribed in a total volume of 20 μL using SuperScript IV VILO Master Mix (Invitrogen, Ref.11756050) according to the manufacturer’s instructions. The cDNA was stored at − 80 °C until further use. Reverse transcription reactions were diluted to 1/10 in RNAse free water and 3 µL of the diluted cDNA was used for each quantitative PCR. qPCR was carried out using sequence-specific primers on a StepOnePlus RealTime PCR System (Applied Biosystems/Thermo Fisher Scientific) in 96-well plates with SYBR Green dye (Platinum SYBR Green qPCR SuperMix-UDG, Thermo Scientific, Ref. 11733-046). All experiments were run in triplicate, and the results were normalized to GAPDH gene (mKIF11-F1 and R1, Ref 804490-689.15 and mGAPD-HF1 and R1, Ref 804490-955.34, Eurogentec).

### Lens-free microscopy and holographic reconstruction

Lens-free acquisitions were performed with a Cytonote microscope (Iprasense, Montpellier, France). This setup is inspired by the lens-free imaging system described in Ref.^30^, which was modified to perform continuous monitoring of cellular cultures inside an incubator at a controlled temperature and humidity^2^. Illumination is provided by a red LED (647 nm, FWHM 13 nm). The light passes through a 150 µm pinhole at a distance of approximately 5 cm from the sample. The CMOS sensor is in contact with the cell culture recipient at a distance of ∼ 1.5 mm (Supplementary Fig. S1).

In the absence of optics, the sample image is obtained by a reconstruction algorithm. For lens-free microscopy, the reconstruction problem is usually formulated under the first Born approximation^31^, which restricts the solutions to low scattering objects. Under this approximation light transport is described by the Fresnel propagator which can be analytically inverted. This propagator applied to the field of light in the sensor plane allows estimating the field of light just after the object plane. However the reconstruction problem remains ill-posed since the lens-free setup, in the absence of a reference arm, does not record completely the field of light in the sensor plane but only its intensity. Yet the lack of phase information does not prevent from retrieving the sample image. The problem can be tackled with the following inverse problem approach. Light is described as a complex scalar field and its propagation from one plane to another is obtained using a convolution kernel *h*_*Z*_. Therefore, if *A*_*0*_ is the complex field after the sample, the field *A*_*Z*_ in the detector plane at the distance *Z* from the object is:

1 $$ A_{0} = A_{Z} *h_{Z} , $$

where ‘*’ is the convolution operator. Here, we used a kernel *h*_*Z*_ derived from Fresnel theory:

2 $$ h_{Z} = \left( \frac{1}{iZ} \right)e^{{\frac{{i\vec{r}^{2} }}{\lambda Z}}} $$

i being the imaginary unit and $\vec{r}$ the spatial position in the horizontal plane. In the following, Z is assumed to be known. In practice, it is estimated with a precision of approximately 10 μm by inspecting Z-stack of back-propagated measurements. The convolution of Eq. (1) can be explicitly written as:

3 $$ A_{Z} \left( {\vec{r}^{^{\prime}} } \right) = \smallint d\vec{r}A_{0} \left( {\vec{r}} \right) \cdot h_{Z} \left( {\vec{r}^{^{\prime}} - \vec{r}} \right). $$

The quantity measured by a standard detector is the intensity of the light field: $I_{Z} = \left| {A_{Z} } \right|^{2}$, where the modulus of $A_{z}$ is known but not its phase $\varphi_{Z}$. Whatever the phase $\varphi_{Z}$, $A_{Z} = \sqrt {I_{Z} } .e^{{i\varphi_{Z} }}$ and the corresponding field $A_{0}$ at the sample plane, $A_{0} = \left( {\sqrt {I_{Z} } .e^{{i\varphi_{Z} }} } \right)*h_{ - Z}$ would perfectly match the data. The inverse problem approach consists here in finding the phase $\varphi_{Z}$ so that $A_{0} \left( {\varphi_{Z} } \right)$ minimize the total variation (TV) criterion $\varepsilon \left( {\varphi_{Z} } \right)$:

4 $$\varepsilon \left( {\varphi_{Z} } \right) = \smallint d\vec{r}\sqrt {\varepsilon + \lvert\frac{{\delta A_{0} \left( {\vec{r}} \right)}}{\delta x}\rvert + \lvert\frac{{\delta A_{0} \left( {\vec{r}} \right)}}{\delta y}}\rvert ,$$

where ε is a small valued coefficient (10^–4^) used to make Eq. (4) differentiable and prevent division by 0 in the gradient derivation presented afterwards. The second term is a *L*_*1*_-norm applied to the fields ($\frac{{\delta A_{0} \left( {\vec{r}} \right)}}{\delta x} , \frac{{\delta A_{0} \left( {\vec{r}} \right)}}{\delta y}$) which promotes sparsity. The optimization is performed using the non-linear conjugate gradients iterative method as described by the following pseudocode:

1. initialization $$k \leftarrow 0,{ }D^{\left( k \right)} \leftarrow 0,{ }\varphi_{Z}^{\left( k \right)} \leftarrow 0$$
2. $$k \leftarrow k + 1$$
3. computation of gradient $\nabla \varepsilon^{\left( k \right)}$, of advancement direction $D^{\left( k \right)}$ and advancement step $\sigma^{\left( k \right)}$
4. phase update: $$\varphi_{Z}^{\left( k \right)} \leftarrow \varphi_{Z}^{{\left( {k - 1} \right)}} + \sigma^{\left( k \right)} \cdot D^{\left( k \right)}$$
5. test of convergence, if not reached go back to 2/

The advancement direction $D^{\left( k \right)}$ is obtained by conjugating the gradient $\nabla \varepsilon^{\left( k \right)}$ with the previous direction of advancement $D^{{\left( {k - 1} \right)}}$, using Hestenes-Stiefel formula^32^. The advancement step $\sigma^{\left( k \right)}$ is obtained by developing the TV criterion at order 2 in the advancement direction (Hessian computation) and by minimizing a second-order polynomial of variable $\sigma^{\left( k \right)}$. For the test of step 5/, we simply stop the algorithm when the number of iterations reaches 15.

Supplementary Fig. S2 illustrates the holographic reconstruction process described here above. Supplementary Fig. S2a shows the full field of view of a raw acquisition of culture of HeLa cells. Based on this image the holographic reconstruction produces a phase image of the cells as shown in Supplementary Fig. S2c. The reconstructed phase image exhibits locally wrapped phase artefacts corresponding to cells that induce a phase shift exceeding + π. In order to perform a simple phase unwrapping, we detect the contiguous pixels with phase value below 0, and set these pixels to + π (Supplementary Fig. S2e).

### Cell-tracking

We used the Trackmate algorithm, an open source Fiji plugin for the automated tracking of single particles^33^. The Trackmate Fiji plugin guides the user through several stages of the cell-tracking algorithm, namely a cell detection stage, a cell tracker stage and several filters applied to the cell detections and the computed tracks. We used the following parameters: the estimated blob diameter was set to ~ 15 pixels, the detector threshold was set to 0.7, the linking maximum distance was set to 15 pixels (~ 25 µm), the gap-closing max distance was set to 15 pixels, gap closing max frame gap to 5 and the filter number of spots on tracks was set to 10. At the end of the cell-tracking process, results are generated in the form of text files. The output file ‘Spots in tracks statistics’ contains a table listing all detected cells with their respective positions in the acquisition, their frame number and their track number.

### Cell dry mass calculation

The phase recovered from lens-free microscopy is proportional to the density and thickness of the specimen layer^34^. A relation is defined between the phase shift and the optical path difference (OPD), which quantifies the integral of the sample object refractive index difference with respect to the surrounding medium along the optical path:

5 $$ \varphi_{shift} \left( {x,y} \right) = \varphi \left( {x,y} \right) - \varphi_{medium} , $$

$$ OPD\left( {x,y} \right) = \lambda \cdot \frac{{\varphi_{shift} \left( {x,y} \right)}}{2\pi }\mathop \smallint \limits_{0}^{h} \left[ {n\left( {x,y,z} \right) - n_{medium} } \right]\delta z, $$

where $n$ is the local sample refractive index, $n_{medium}$ is the surrounding medium refractive index, z is the position along the optical axis, h is the thickness of the sample object and λ is the illumination wavelength. The *OPD* values can be integrated over the total projected area *S* of the cell. Here we used the optical volume difference ($OVD$) denomination introduced in Ref.^35^ to define this integral. It is expressed as a unit of volume in μm^3^:

6 $$ OVD = \mathop \smallint \limits_{S}^{{}} OPD\left( {x,y} \right)\partial x\partial y. $$

To determine the cell area *S*, we used a seeded growing segmentation algorithm controlled by a single parameter, a phase threshold value which delineates the separation between the background and the cell area (see Supplementary Fig. S3). A relationship between the phase shift measurement and the cell mass has been defined in Refs.^36,37^ and can be used to convert $OVD$ to cell dry mass ($CDM$) measurements, the mass of the cellular content except water. Under our notation, this relationship is simply given by:

7 $$ CDM = \frac{OVD}{\alpha }, $$

where α is the specific refractive increment which relates the refractive index change to increase in mass density. There is a variety of substances within a cell. However the specific refractive index of these substances falls with a narrow range and Barer^36^ defined an α constant of 0.18 µm^3^ pg^−1^ for most eukaryotic cells, taking into account not only proteins, but also lipids, sugars, and nucleic acids.

To estimate the precision of the *CDM* measurement obtained by means the lens-free microscope we compared the cell measurements with two other techniques: digital holographic microscopy (DHM) and quadriwave lateral sheering interferometry (LSI). The measurements obtained with lens-free microcopy correlate linearly with the measurements obtained with DHM and LSI (see Supplementary Fig. S4). It suggests that the lens-free microcopy setup can be considered as a quantitative phase imaging technique for the measurements of adherent cells. The values measured with lens-free microcopy are however under-estimated, they must be corrected by a factor of ~ 1/0.65. This is due to the sparsity constraints used in the holographic reconstruction algorithm (see Eq. 4) which reduces overall the phase signal. According to a methodology and the dataset described in Ref.^34^, we can estimate the precisions of the *CDM* measurements obtained with the lens-free microscopy setup used in this study to be about 35 pg.

### Fourier transform and other spectral analysis algorithmes

The *Fourier transform* algorithm allows real-time *spectral analysis* of the dry mass and help us to find the spectrum of possible periodic signals. We compute the Fourier transform over the duration T(j) of our measurement of the dry mass of each cell j during interphase. T(j) is defined to be the time between the two local spikes of dry mass^2^ that correspond to the successive divisions that delimit the interphase or, for incomplete cycles, the time from the first local spike of dry mass until tracking of the cell is lost. After removing the background, the signal was convoluted with a sine window function that vanishes at the limiting times. The FT was then performed for each frequency with an increment equal to df(j) = 1/(T(j)*M) depending on cell j, where M is an integer corresponding to the chosen resolution in frequency sampling. We fix M to be equal to 25 for all the samples. The set of all transforms was analyzed statistically by computing the averaged absolute amplitude, as well as the standard deviation, in each frequency bin df = n_f_/(T_max_*M). The averaged FT was performed using bins defined by the longest time duration T_max_ = max{T(j)}, and n_f_ is an arbitrary positive integer, defining the size of the bin. In general, we take n_f_ = 1 or 2. The averaged absolute amplitude was properly normalized in order to take into account the different bin sizes and lengths of the traces.

### Repeatability of measurements of the period of the rhythm

We have repeated the control experiments with more than 30 runs (all cells lines combined; see Supplementary Fig. S5 for an example). The experiments comparing the effect of drugs were repeated two or three times independently for each drug concentration, type of cell (MEF and HeLa) and siRNA. The experiments testing temperature effects were repeated three times independently for each temperature and cell-type (HeLa, CHOK1 and their mutants tsTM18, tsTM3). The experiments on cell-cycle inhibitors were each repeated at least three times independently. In each experiment, we followed a number of individual cells varying from 300 to 2400.

## Supplementary Information

Supplementary Video 1.

Supplementary Video 2.

Supplementary Information.

## Data Availability

Data are available in the main text and in the supplementary materials. Image and trajectory data can be supplied by request to the authors.
