# Supplementary material for: A new ultradian rhythm in mammalian cell dry mass observed by holography
Source: Sci Rep. 2021 Jan 14;11:1290. doi: 10.1038/s41598-020-79661-9 (PMC7809366; doi:10.1038/s41598-020-79661-9)
Supplement: Supplementary file 3 — Supplementary Information. [file 41598_2020_79661_MOESM3_ESM.docx]

**A new ultradian rhythm in mammalian cell dry mass observed by holography**

Lamya Ghenim^1,2*^, Cédric Allier^3^, Patricia Obeid^2^, Lionel Hervé^3^, Jean-Yves Fortin^4^, Maxim Balakirev^2^, Xavier Gidrol^2*^

^1^Univ. Grenoble Alpes, CNRS, CEA, INSERM, IRIG, Biomics F-38000 Grenoble, France.

^2^Univ. Grenoble Alpes, CEA, INSERM, IRIG, Biomics F-38000 Grenoble, France.

^3^Univ. Grenoble Alpes, CEA, LETI, F-38054 Grenoble, France.

^4^Laboratoire de Physique et Chimie Théoriques CNRS UMR7019, Université de Lorraine
1 Boulevard des Aiguillettes, BP 70239 54506 Vandoeuvre-lès-Nancy Cedex.

^*^Correspondence:

[lamya.ghenim@cea.fr](mailto:lamya.ghenim@cea.fr) (L. G.)

[xavier.gidrol@cea.fr](mailto:xavier.gidrol@cea.fr) (X. G.)

TABLE OF CONTENTS

**Supplementary Figures S1 to S10 Page 2 to 12**

**Supporting Information**


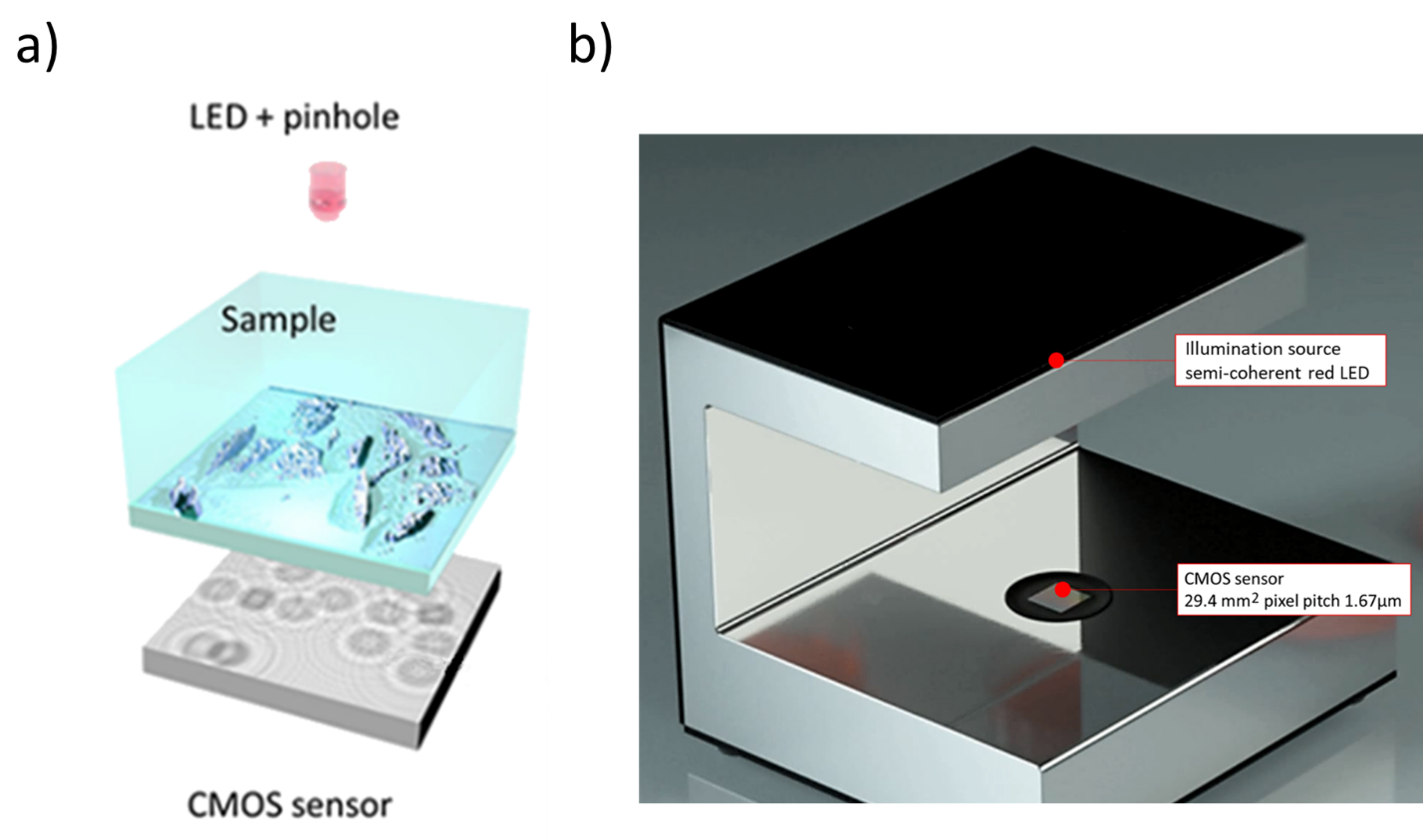


**Supplementary Figure S1.** Lens-free Microscopy. (a) Schematic of the lens-free microscopy acquisition setup. The CMOS sensor is put in contact with the Petri dish and records the holographic pattern resulting from the interference between the partially coherent incident light and the light scattered by the cells. (b) The lens-free microscope is the Iprasense Cytonote. It features a CMOS image sensor with a pixel pitch of 1.67 μm and an imaging area of 6.4 mm x 4.6 mm. Illumination is provided by a red LED along with a 150 µm pinhole placed at a distance of approximately 5 cm from the sample.


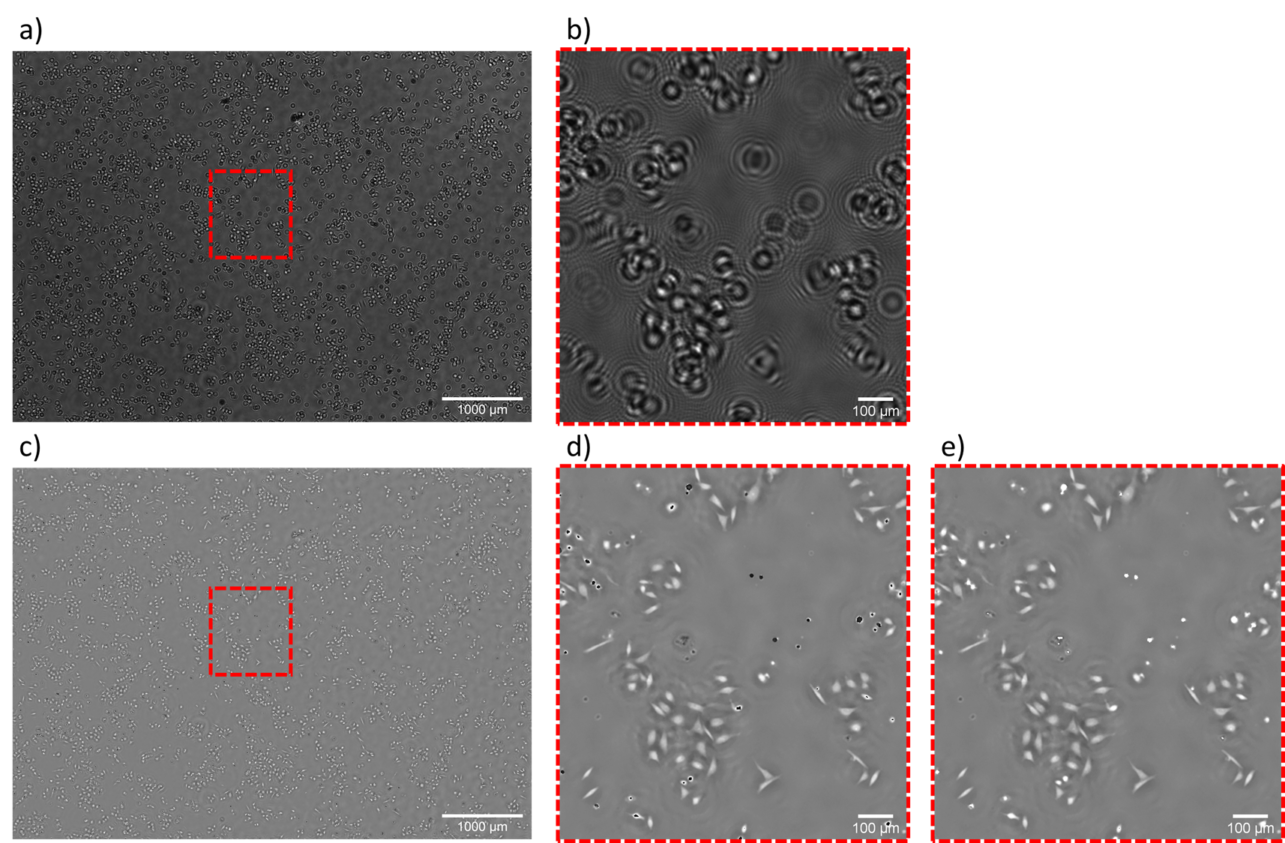


**Supplementary Figure S2.** Holographic Reconstruction Process. (a) Raw acquisition of HeLa cells in culture by means of lens-free microscopy (full field of view of 29.4 mm^2^). (b) Detail of (a). (c) Reconstructed phase image (full field of view of 29.4 mm^2^). (d) Detail of (c). (e) Phase image obtained after phase unwrapping. To be compared with (d), before unwrapping.


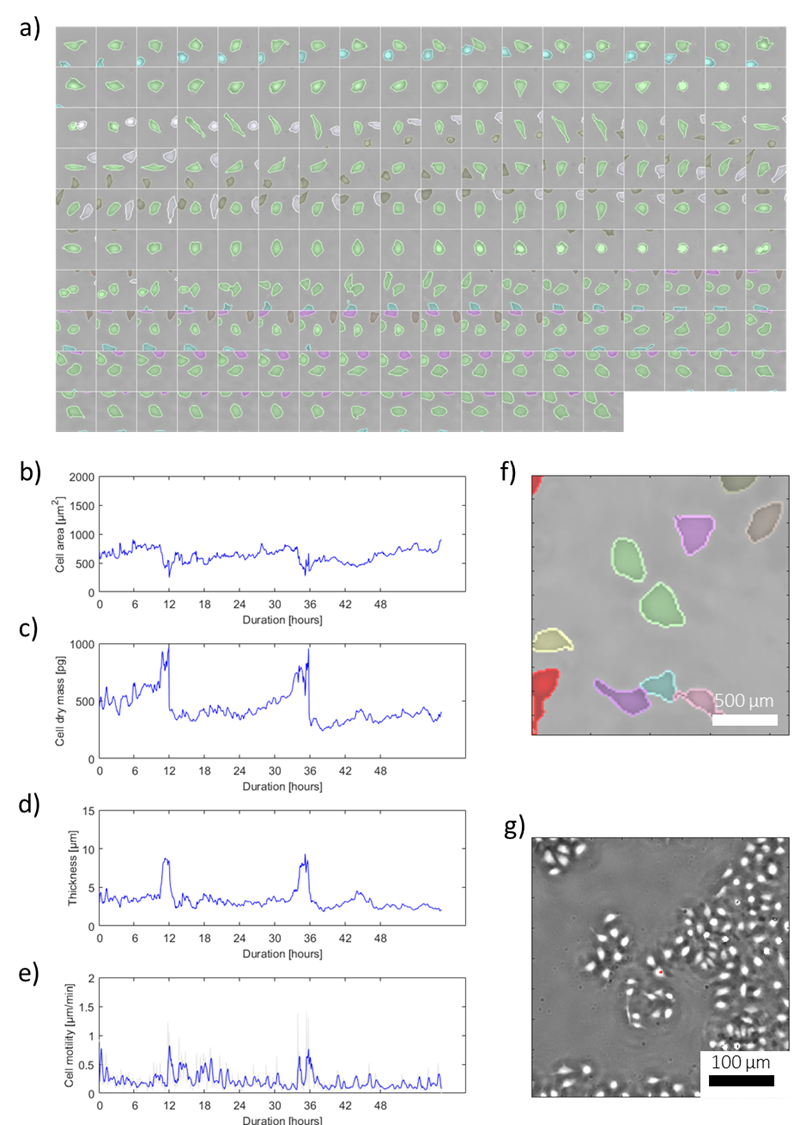


**Supplementary Figure S3.** Data Analysis of a 2.4-days Cell Trajectory (5 min. frame interval, 710 frames). (a) Cell segmentation performed on the time-lapse acquisition as a function of time (20 minutes interval). The cell of interest is shown with a green overlay. Each cropped image is 75x75 µm^2^. The two cell divisions are shown with red circles. (b) Plot of the cell area as a function of time. (c) Time evolution of the cell dry mass calculated from the integral of the phase over the segmented cell area according to Eqs. (5-7). (d) Plot of the average cell thickness calculated as a function of time. The cell thickness exhibits sharp peaks corresponding to cell divisions (red circles in (a) and red lines in (b-c-d-e)). (e) Plot of the cell motility as a function of time. (f) Segmentation results corresponding to the last frame of the track. The image is 200x200 µm^2^ centered on the cell of interest. (g) 800x800 µm^2^ cropped reconstructed phase image of the last frame. The cell of interest is in the middle with a red spot.


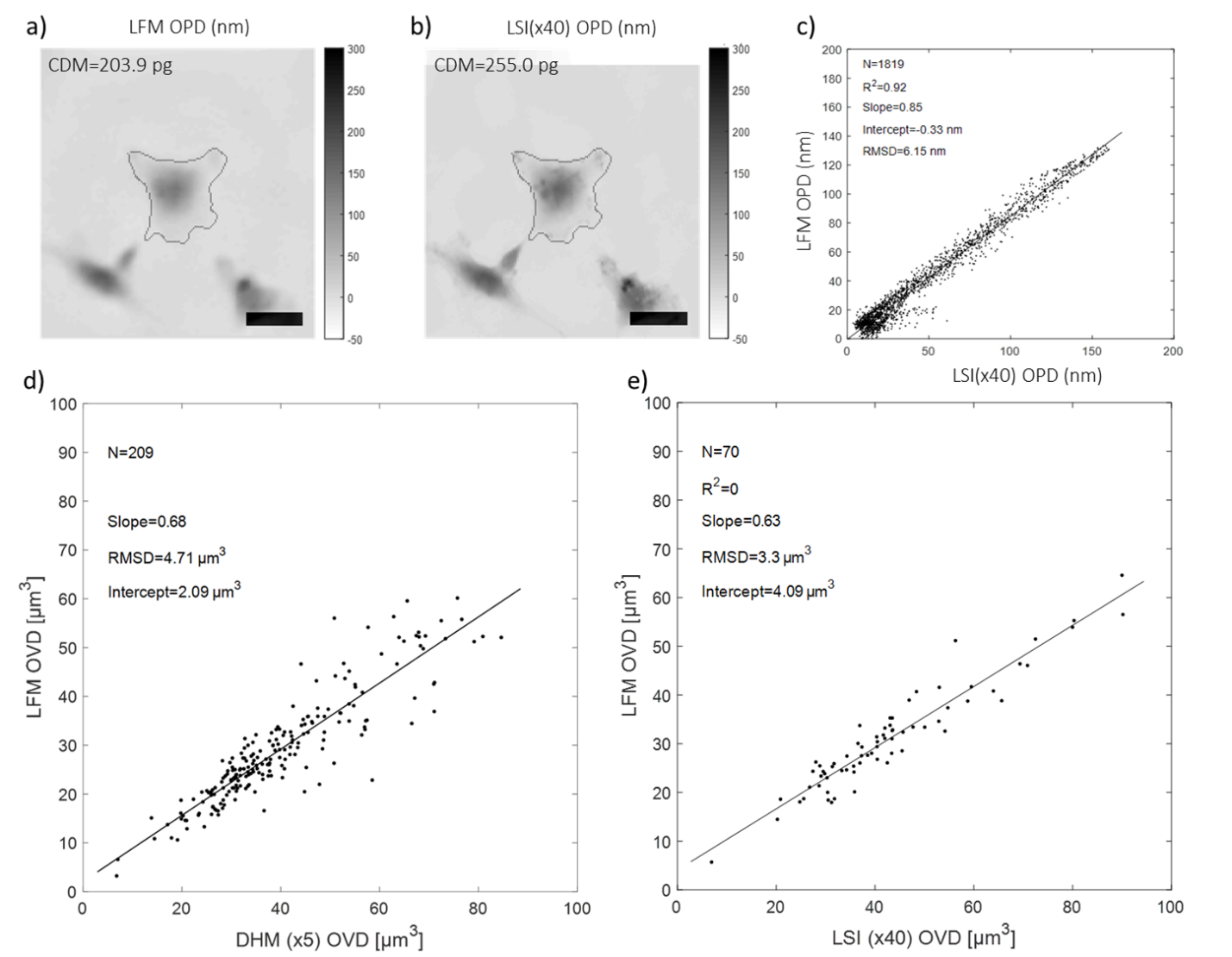


**Supplementary Figure S4.** Cell Dry Mass Measurement. (a) OPD (optical path difference) map (nm) of a fixed COS-7 cell obtained with lens-free microscopy (LFM). Scale bar is 25 µm. The contour of the cell segmentation area is shown in black. (b) OPD map obtained for comparison with quadriwave shearing lateral interferometry (magnification x40, wavefront sensor SID4Bio Phasics, Saint-Aubin, France). (c) Pixel to pixel comparison between the OPD maps (within segmented cell area). The results of the linear regressions are indicated with values of slope, intercept, coefficient of determination (R^2^) and root-mean-square deviation (RMSD). (d) and (e) Pair-wise comparisons of OVD (optical volume difference, see Eq. 6 in manuscript) measurements of fixed COS-7cells. (d) Comparison between lens-free microscopy and digital holography (magnification x5, DHM T-10105 Lyncée Tec, Lausanne, Switzerland). (e) Comparison between lens-free microscopy and quadriwave shearing lateral interferometry. The linear regression fitting curves are plotted in black. The results of the linear regressions are indicated with values of slope, intercept, coefficient of determination (R^2^) and root-mean-square deviation (RMSD). N refers to the number of cell measurements per comparison.


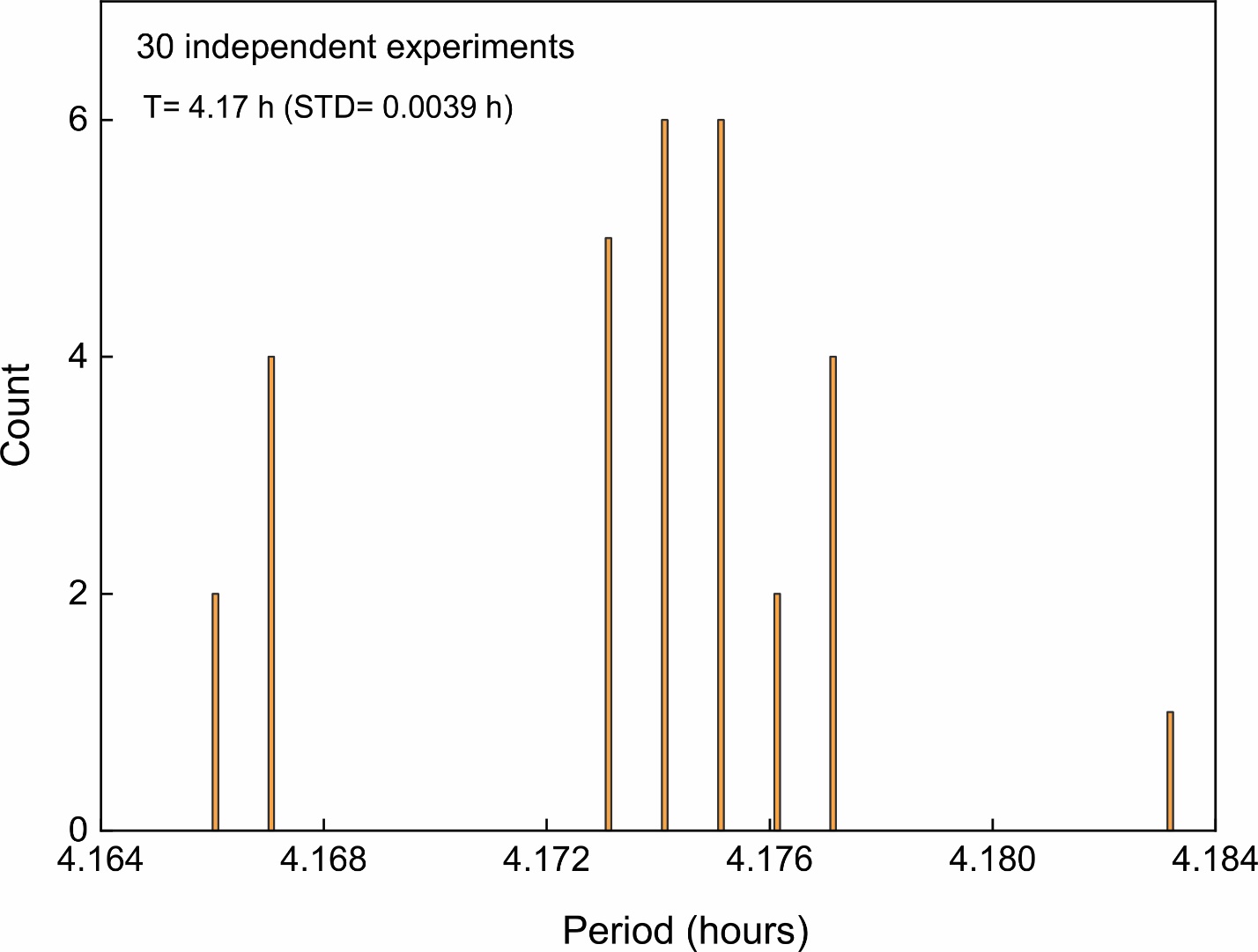


**Supplementary Figure S5.** Histogram of the period of the rhythm as measured in 30 independent experiments. The average value and standard deviation are T=4.17h and 0.0039h respectively.


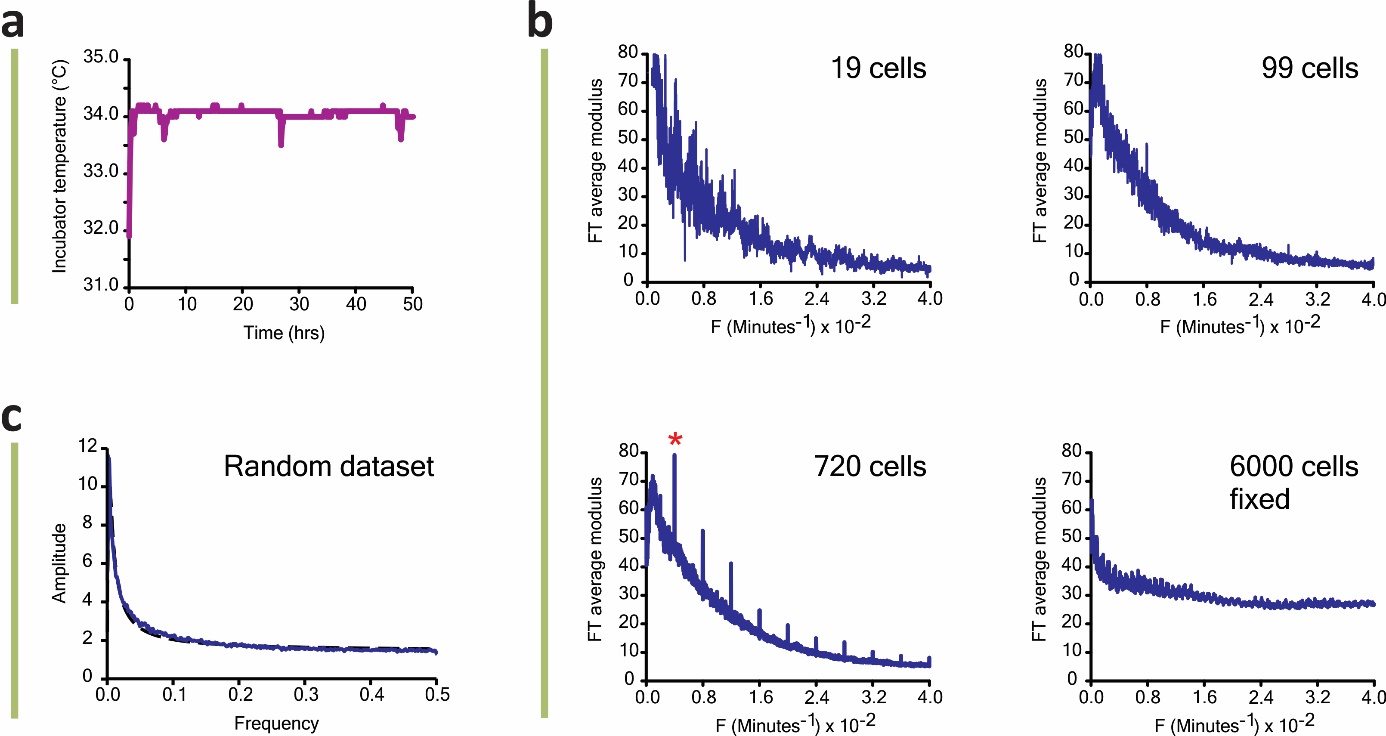


**Supplementary Figure S6.** Test Experiments. (a) The incubator temperature as a function of time; no periodicity is seen. (b) Average FT for increasing numbers of MEF cell trajectories. Periodicity appears only as the number of cells increases beyond 100. Bottom right: 6000 fixed cells show no rhythm. (c) FT analysis of a random dataset detects no periodicity.


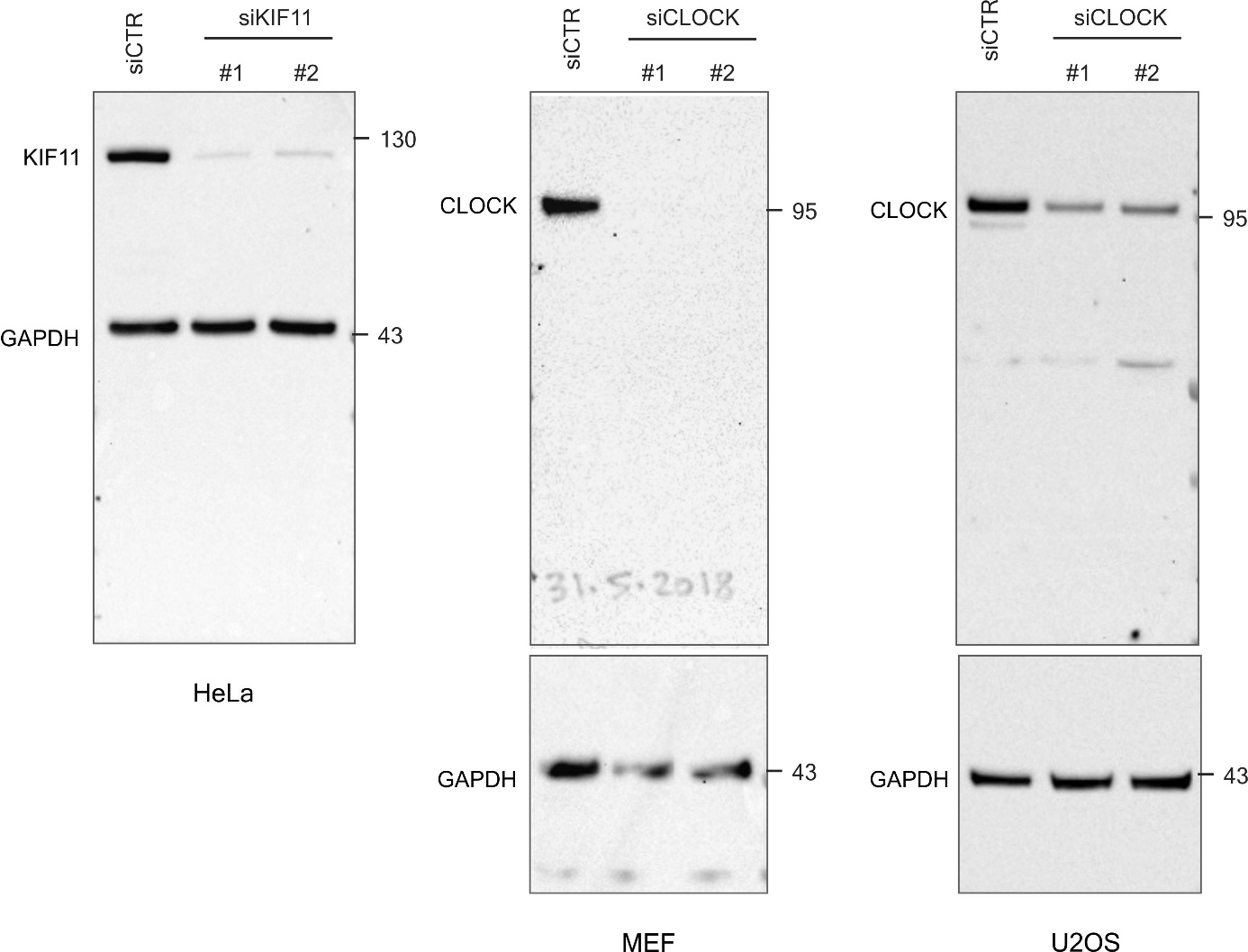


**Supplementary Figure S7.** Original, unprocessed Western blots for Fig. 3 confirm the inhibition of KIF11 expression (the effect of two specific siRNAs is compared to non-specific siCTR) and show the inhibition of CLOCK expression by siCLOCK 1 and 2 in MEF and U2OS cells.

**Supplementary Figure S8.** Reproducibility of FT Measurement. Two independent experiments with CHO-K1 cells.

**Supplementary Figure S9.** Characterization of the Inhibitor Cytotoxicity. Dose response curves for Actinomycin D (ActD), Cycloheximide (CHX), MG132 and Bortezomib (Btz) in HeLa (upper row) and MEF (lower row). The percentage of apoptotic cell was measured with CellEvent reagent as described in Materials and Methods. Diagrams Drawn with the software package R from the R Core Team^29^.

**Supplementary Figure S10. The Rhythm Inhibition by Bortezomib does not Arrest the Ongoing Cell Cycle.** HeLa cells treated with 5 nM Bortezomib are still cycling as seen from the increase in cell number (left). The mean cell cycle time is 19.2 hours (middle), whereas the rhythm is completely suppressed (right).
